# De-Identification Mechanism of User Data in Video Systems According to Risk Level for Preventing Leakage of Personal Healthcare Information

**DOI:** 10.3390/s22072589

**Published:** 2022-03-28

**Authors:** Jinsu Kim, Namje Park

**Affiliations:** 1 Department of Convergence Information Security, Graduate School, Jeju National University, Jeju-si 63294, Korea; kimjinsu@jejunu.ac.kr; 2 Department of Computer Education, Teachers College, Jeju National University, Jeju-si 63294, Korea

**Keywords:** de-identification of persons, image processing, face recognition, access control, crime database, IoT CCTV environment

## Abstract

A problem with biometric information is that it is more sensitive to external leakage, because it is information that cannot be changed immediately compared to general authentication methods. Regarding facial information, a case in which authentication was permitted by facial information output by a 3D printer was found. Therefore, a method for minimizing the leakage of biometric information to the outside is required. In this paper, different levels of identification information according to the authority of the user are provided by the de-identification of metadata and face information in stages. For face information and metadata, the level of de-identification is determined and achieved according to the risk level of the de-identified subject. Then, we propose a mechanism to minimize the leakage path by preventing reckless data access by classifying access rights to unidentified data according to four roles. The proposed mechanism provides only differentially de-identified data according to the authority of the accessor, and the required time to perform the de-identification of one image was, on average, 3.6 ms for 300 datapoints, 3.5 ms for 500 datapoints, and 3.47 ms for 1000 datapoints. This confirmed that the required execution time was shortened in proportion to the increase in the size of the dataset. The results for the metadata were similar, and it was confirmed that it took 4.3 ms for 300 cases, 3.78 ms for 500 cases, and 3.5 ms for 1000 cases.

## 1. Introduction

The COVID-19 outbreak has caused significant damage in our lives, and, in addition to the direct damage caused by its occurrence, the collateral damage resulting from the difficulty of making direct contact whilst avoiding the transmission of the disease is noteworthy [1,2,3,4]. There is now growing demand for contact-free technology, which does not require face-to-face contact. To maintain an environment where direct contact is avoided by implementing social distancing, it is crucial to authenticate that the users accessing the system are properly authenticated [5,6,7].

The typical authentication method is the basic ID/password method, which means generating a specific string that only the user knows and authenticating the user with its input. As such, authentication that is performed with only one authentication means is being threatened, and many studies are underway to block access by third parties through the more thorough authentication of users [8,9,10,11]. Fingerprint-based biometrics as well as OTPs (one-time passwords), which are mainly used for two-factor authentication, are being used [12,13,14].

Biometric authentication, which authenticates users based on their biometric information, can be applied by fingerprints, irises, and faces. Among them, in the case of dual facial recognition, China’s Skynet Project has built a system that can find specific individuals by using surveillance camera footage taken in real time via an imaging system and a database that records suspected criminal information. Furthermore, in Xiamen City, Fujian Province, China, which introduced an image-based facial recognition system, the effect was a 30% reduction in pickpocket crimes, thereby proving its effectiveness [15,16]. However, the drawback is that, aside from the effectiveness of the system, it is difficult to introduce it suddenly due to the privacy infringement of the users who are being recorded [17,18,19]. Such disadvantages can be directly related to the problem that biometric information utilization in smart cities may be difficult due to malicious actions using leaked biometric information as well as the invasion of personal privacy.

In particular, the database for tracking criminal suspects uses not only general metadata such as the suspect’s gender, age, and identification number, but also biometric information such as the subject’s face or fingerprint, and carries the risk of becoming a path for biometric information leakage. Therefore, biometric information recorded in the database must be processed so that it cannot be used by a third party, and in the system proposed by this paper, it is prevented from being leaked indiscriminately by applying different processing techniques according to the risk level. In addition, by dividing the access requester into four roles and by varying the de-identification stage of the metadata and biometric information provided according to each role, only the data suitable for the access requester are provided, and other information cannot be identified. This aims to reduce the possibility of leakage. This paper analyzes related technology and research trends in Section 2 and outlines the proposed risk-level-based de-identification mechanism in Section 3. After that, in Section 4, the functions applied to the mechanism are described and the performance results are analyzed.

## 2. Related Studies

### 2.1. Image Tracking Systems

An image tracking system generally refers to a system used to track the travel path of an object recorded by CCTV (closed-circuit television) in a connected video system [20,21]. The primary purpose of image tracking systems is to track the travel path of a specific individual; they are used for purposes that require the need to continuously monitor or detect the behavior of a specific individual in real time or in the future [22,23]. The video surveillance system in China can serve as an example. The video surveillance system “Skynet (Tiān wǎng)” nationally operated by the Chinese government is a system that can track objects recorded by CCTV installed in the city center [24,25]. The Chinese government has touted that the system can identify individuals based on the characteristics of each object and has a facial recognition rate of up to 99.8% [26,27]. In addition, schools in China are taking advantage of a variety of facial recognition devices, such as facial recognition and the thermal measurement of students via imaging devices. However, object analysis, which has made great progress because of imaging systems, is controversial due to the issue of privacy infringement, and therefore a measure to ensure privacy for irrelevant objects is required.

### 2.2. Crime Databases

The forensics field in Table 1 includes facial information for tracking criminals or missing persons [28]. Not only that, it can be seen that personal information such as counterfeit documents and vehicle information is also contained. Although the personal information of a suspect is essential for criminal investigation, it must be protected until the suspect is determined to be an actual criminal, and the criminal record must be made available to the person responsible for investigating the case. The system we propose enhances the security of personal information by varying the level of de-identification based on the risk level of suspects; hence, it is designed to limit the disclosure of information pertaining to low-risk suspects.

### 2.3. Research Trends in Related Fields

Our proposal introduces a measure to protect privacy via the prevention of the indiscriminate disclosure of information, by limiting the information accessible to authorized users depending on their role via access control based on facial recognition and by varying the amount of accessible user information that is disclosed depending on the risk level. In this section, we analyzed the research trends regarding privacy protections applied to video surveillance systems.

The threat of privacy infringement caused by video surveillance systems begins with extracting sensitive areas contained in the video footage. Sensitive areas contain information that can be inferred to pertain to a specific individual, which may include biometric information such as facial information, skin color, and hair, as well as personal items such as bags and clothes. The de-identification or anonymization of the facial area is a technology that is being studied extensively to make it difficult to infer the identity of specific objects through their faces. Ling limited the information that can be accessed by users depending on their authority by distinguishing between blurred public streams and private streams, mentioning the need for an information protection system that can solve the challenges of anonymization, image preservation, recoverability, and compressibility for data security in video surveillance systems [29].

Regarding the anonymization technology of the facial area, research on the architecture based on GAN (generative adversarial networks), which means hostile neural networks, is being actively performed. Yifan proposed a framework called PP-GAN (Privacy Protective-GAN), which produces the result of de-identification with similarity to a single input, pointing out that the majority of the typical pipelines used for the de-identification of the facial area rely on the k-same framework, while the k-same framework shows poor results in terms of efficiency and visual quality [30,31]. Alakh proposed a higher-resolution facial anonymization technology by presenting a framework called EPD-Net to preserve biological and non-biological features at high resolution, mentioning the need to preserve structural integrity and the required non-biological information at a high resolution [32,33,34].

Prior research has focused on the protection of privacy, aiming at de-identifying sensitive areas captured by the video surveillance system. However, there are cases where tracking is required based on biological or non-biological information, such as criminals or missing persons [35,36]. In this regard, it may be necessary to infer a specific object by adjusting the degree of de-identification depending on the object.

## 3. Proposal for a De-Identification Mechanism Based on Risk Levels

**Figure 1:**
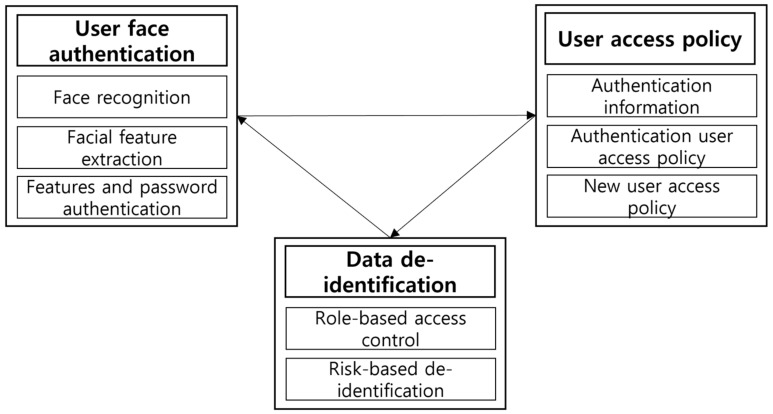
The de-identification mechanism model of the user data in the imaging system.

The purpose of this study is to prevent the indiscriminate exposure of identity by restricting the disclosure of information according to the role of an unspecified number of users accessing personal information databases, including personal information and face information. In particular, as IoT technology develops, in recent healthcare, not only face-to-face services but also home care services are being developed. Therefore, the subject’s personal information can be moved online and recorded in a database that intensively manages it. However, it is not necessary for a user who accesses a database in which personal identity is recorded to access all the data, which may be a factor that causes the leakage of personal information. Therefore, there is a need for a method that allows access to limited data based on the role of the user who accesses the database. In this paper, two-step authentication is implemented based on the facial information of the user accessing the database, and role-based access control that provides only limited identity information according to the role of the authenticated user is implemented. The roles are broadly divided into four types: chief administrator, investigator, server operator, and guest. The chief administrators are granted access to all records in the system, while investigators are only granted access to the criminal database. The server operators are partially granted access to the criminal database to prevent excessive information leakage, and no data are provided to guest users who are not logged in. Privacy infringement factors can be reduced by setting up the data recorded in the crime database by varying the degree of de-identification depending on the risk level of a specific object and by blocking access to data for users who pose an extremely low risk so that the higher the risk level, the easier the specific individual is to be identified. Figure 1 shows the model structure of the de-identification mechanism of the imaging system user data that we propose.

**Figure 2:**
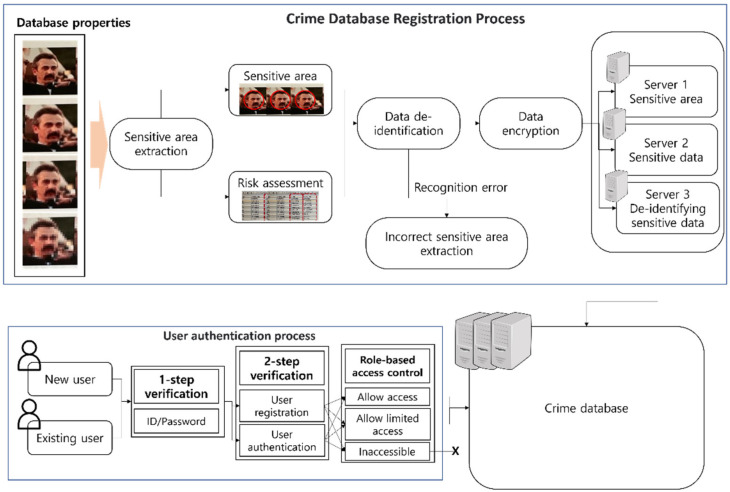
A diagram of the de-identification mechanism of user data in the imaging system.

The mechanism for implementing the de-identification of user data in the imaging system we advocate in this paper proposes an overall structure that can be utilized in a criminal database. The processes undertaken by the mechanism we propose can be divided into two parts: the registration process for the crime database, and the process of providing appropriate data via user authentication. Figure 2 shows the overall structure of the mechanism we propose.

First, the registration process for the crime database begins with the process of registering the images of suspects in the system. The procedure for extracting the sensitive areas from the images registered in the system is performed, and the sensitive information at this time is limited to the facial area of the specific individual to be registered. The degree of the de-identification of the sensitive area is determined by the risk assessment of the extracted sensitive information registered by the system administrator. However, if the sensitive information is not correctly recognized, error handling will proceed, as it is incorrect sensitive information. Sensitive information that is successfully recognized is recorded in the criminal database on the server after data encryption. The crime database is configured with a server for sensitive areas that has sensitive areas that are not in a de-identified state; a server that records the sensitive information of metadata that has not been de-identified and can infer a specific individual, including their name, age, gender, etc.; and a server that records de-identification information for the facial area and de-identification information for metadata.

The user authentication process is largely composed of the following: a one-factor authentication process consisting of an ID and password and a two-factor authentication process using facial area verification and a password. Users authenticate themselves through one of these means and acquire access to data that have not been de-identified or to de-identified data after being authenticated as legitimate users.

## 4. Detailed Diagram by Module

The mechanism proposed in this paper contains three modules: facial recognition, role-based access control, and the de-identification process. The facial recognition module is in charge of recognizing a facial area, which is sensitive personal information included in the data, when image data including facial information are generated in the process of inputting data by a user. Role-based access control is a module used when a user requests data recorded in a database. When a data request is made by a user, the module is responsible for confirming four defined user roles and providing data of a certain de-identification level according to the user’s role. The de-identification process module refers to a module that substantially de-identifies data according to the user’s role, as determined by the role-based access control. The module receives data pertaining to the object according to the user’s request and determines the level of identification of the user by performing different levels of de-identification based on the risk of the object.

**Figure 3:**
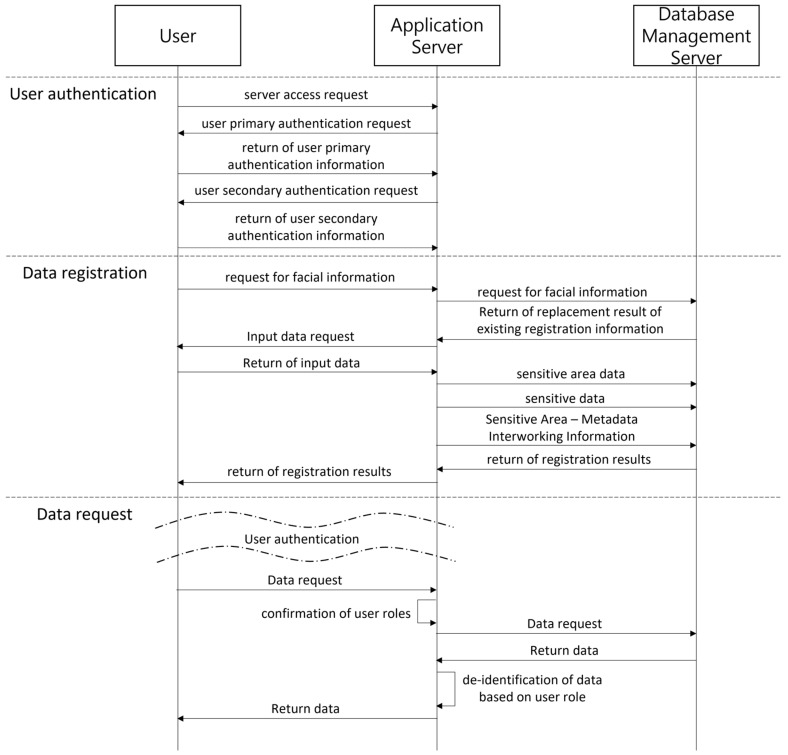
A diagram of the de-identification mechanism of user data in the imaging system.

Figure 3 shows the order of user authentication, data registration, and data request for the proposed mechanism. The main function of the mechanism proposed in this paper is to provide differentiated de-identification data according to the role of the user. Therefore, there are three types: a user authentication process in which a user accesses a server, a data registration process in which a user inputs data to a database, and a data request process in which a user requests data from a database.

### 4.1. Facial Recognition

Both the detection of the facial area in the image for registration and the user authentication in the criminal database are performed by the same process. When attempting facial recognition to authenticate the identity of the user in the system, the user’s identity should not only be recognizable from various directions, including the sides, in addition to the general frontal image, but it should also be recognizable even under the environmental factors of the surroundings, such as the environment of the recorded area and the amount of sunlight. As a measure to recognize a specific individual despite the variables, there is machine learning technique that can gradually improve the accuracy by learning a large number of images.

To analyze patterns, the selection of characteristic parts of the image must be carried out, and, at the same time, the operating speed must also be taken into account. To take into account the operating speed, the designated range must be widened; to show a clearer result for a feature, a range with high similarity must be set. To satisfy this point, the characteristic should be able to be detected based on the result value derived by dividing the intervals into two for a given range and subtracting the sum of the two intervals. In this paper, we applied the Haar cascade algorithm for the detection of captured subjects.

**Figure 4:**
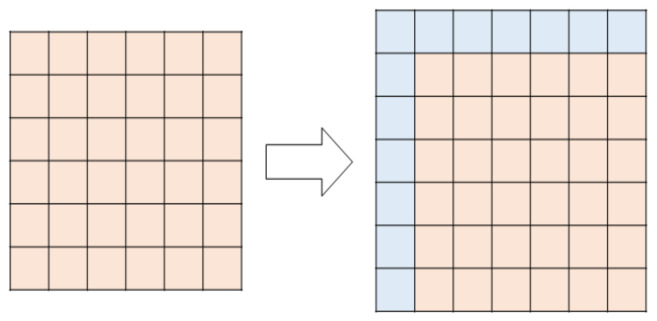
Creation of integral image.

To analyze the pattern of the image, we created an integral image, which has a height and width one value larger than the conventional image. The value of the integral image may vary depending on which area is set, and the input image area is set to f(x, y). Further, when the horizontal axis of the image area to be integrated is expressed as xi, the vertical axis is expressed as y_i_, and the integrated image is expressed as f(x_i_, y_i_); f(x_i_, y_i_) has the same size as f(x + 1, y + 1). Figure 4 shows how an integral image is created.

The calculated value of the integral image varies depending on the size of the area designated to detect the characteristics, and the calculated value is the result value on the lower right of the designated area. After that, a process of calculating the characteristic value of a designated area is performed. When the size of the area designated to obtain the characteristic value of the area is expressed as x_c_ and y_c_, and the characteristic value is expressed as z, Equation (1) shows the process of calculating the characteristic value of a specific area.

(1) $$z=f\left(x_{c},y_{c}\right)-f\left(x_{c},y_{c-c}\right)-f\left(x_{c-c},y_{c1}\right)+f\left(x_{c-c},y_{c-c}\right)$$

**Figure 5:**
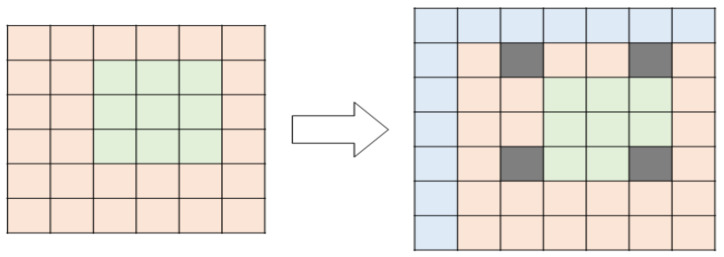
Calculation of the characteristic value of the integral image.

Figure 4 shows the range applied to perform the calculation of the characteristic value of the area designated in the integral image. In the case of calculating the characteristic value of a region, an additional region designation is required for performing integration, rather than including only the region of the initially given image. Therefore, a larger area including the designated area is required. Thus, in Figure 5, it can be seen that the starting point of the region is set as previously described for the x-axis and y-axis of the designated region.

### 4.2. Role-Based Access Control

If the result of performing two-factor facial recognition based on the detected facial area is recognized as a legitimate user, accessible information is provided based on the user’s role. The roles of persons with access to the system are broadly divided into four categories: chief administrator, investigator, server operator, and guest. Table 2 explains each of their roles.

As shown in Table 2, the restrictions on the data given to each role are different. Restricted data are broadly divided into metadata and videos. Metadata refer to information recorded in the criminal database and include the name, age, height, weight, vehicle number, etc. of the subject. Image information refers to the image information of the subject. At this time, the image information is disclosed after being de-identified depending on the risk level of the subject, and details on de-identification will be described in the following section.

**Figure 6:**
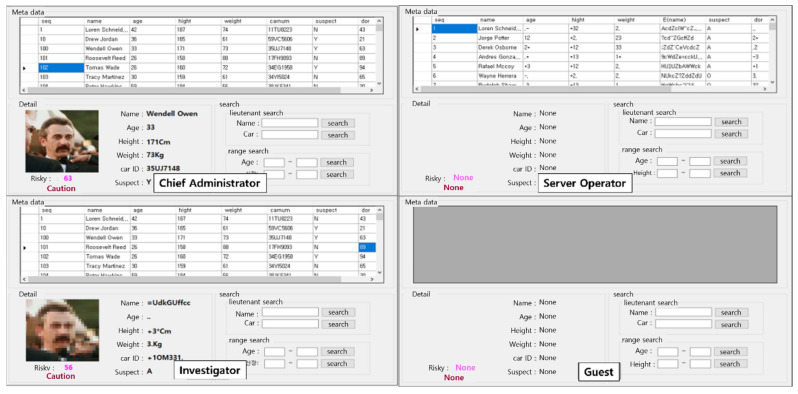
Scope of information disclosure by role.

Figure 6 shows the range of information that can be accessed for each role. The upper left shows the access history of the chief administrator; it can be seen that the information and video recording that belong to the metadata are provided. The lower left shows the range of information that is disclosed to the investigator, and it can be seen that metadata that have not been de-identified and the video information that has been de-identified are given. The figure on the upper right is the data that are given to the server administrator, and only metadata that have been de-identified are given. The figure on the lower right shows the information given to the guest, and it can be seen that no metadata or image information are given.

### 4.3. De-Identification Process

The de-identification process for the subject is divided into the processing of the identification information and the image information processing. In the identified information processing, the identified information is converted into irrecoverable data by performing a hash calculation on the identified information recorded as a character string. The de-identification of identified information is performed for each item to designate and navigate the subsequent range. The converted data can be made public, because it is difficult to restore them to their original state, even if disclosed.

**Figure 7:**
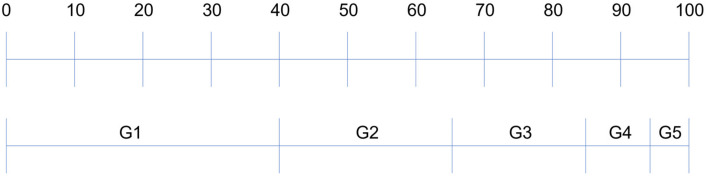
Range of de-identification depending on the risk level.

The following is a description of the de-identification process for image data. The de-identification process for image data yields different levels of de-identification according to the risk level. Accordingly, high-level de-identification is applied to a low-risk subject, making it difficult to identify them; conversely, the degree of de-identification is rendered lower for a high-risk subject, yielding identifiable image information. Figure 7 shows the groups classified according to risk level.

Table 3 shows the terms used to de-identify image information.

As groups classified by risk level should have higher de-identification variable values at low figures, the lower groups have higher figures. The base data applied for risk calculation may vary depending on the application environment and may ultimately have different result values according to weights for the data used for risk calculation. Equation (1) includes values specified according to the risk level to vary the degree of de-identification for each group. Equation (1) arbitrarily sets the range of risk stages, grouping each stage into one group, and assigning weights. In this case, the higher the weight of the group used, the higher the difficulty of de-identification.

(2) $$G=\left\{\begin{array}{c} G1=50\;if\;Risk\;\leq 40\\ G2=10\;if\;Risk\;\leq 65\\ G3=5\;\;\;if\;Risk\;\leq 85\\ G4=3\;\;\;if\;Risk\;\leq 95\\ G5=0\;if\;Risk\;\leq 100 \end{array}\right.$$

In group information configured as in Equation (1), the de-identification degree for the subject registered in the database is determined as shown in Equation (2). Equation (3) shows the calculation result for the difficulty of de-identifying in the case where the risk level of the de-identified subject is less than or equal to 40.

(3) $$D_{R}=\sum_{n+2}^{n=n}G_{n}\;D_{R}=50+10+5=65\;if\;Risk\;\leq 40$$

Equation (3) shows the process of determining the scope for blurring. As blurring progresses, different degrees appear depending on the scope of the designated area, and the wider the blurring progresses, the more blurred the image provided. The value set according to the risk level of the subject is the standard deviation for setting the scope; if the subject is suspicious, the standard deviation is set to 0.4 and the mask is set to 3 × 3, whereas if the subject is not suspicious, the standard deviation is set to 0.8 and the mask is set to 5 × 5, making it possible to create image information that is more difficult to identify.

(4) $$B_{R}=\left\{\begin{array}{c} 0.4\;if\;Suspect\;Status=Yes\\ 0.8\;if\;Suspect\;Status=No \end{array}\right.$$

For the de-identification of the image information based on Gaussian distribution, the de-identification value calculated depending on the previously calculated risk level of the subject and the standard deviation based on the subject’s charge is converted into a blurring variable. Equation (4) shows a mask created for the de-identification of the image information, and a higher value generates image information that is difficult to identify.

(5) $$Mask_{B}=B_{R}*\left(\left(D_{R}-1\right)*0.5-1\right)+0.8$$

**Figure 8:**
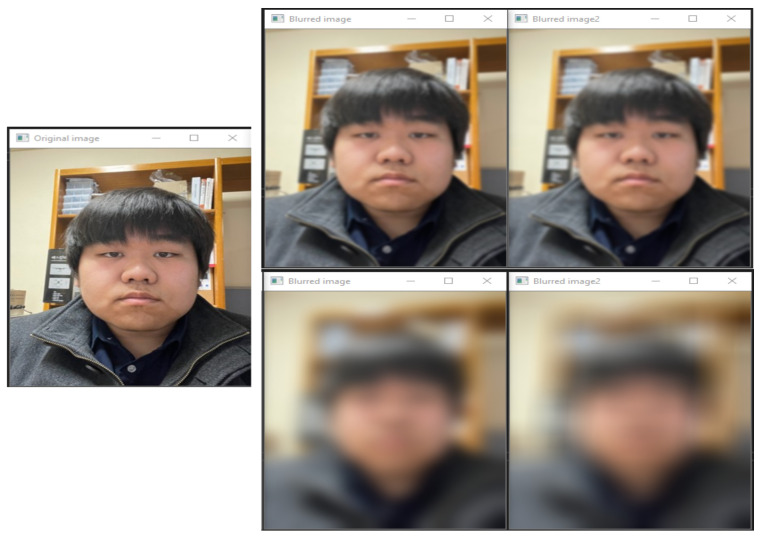
Original image information of the subject to be masked.

When the image is de-identified via a method in which different masking values are given depending on the risk level of the subject and whether they are suspicious, the results are obtained as shown in the figure below. Figure 8 shows the results of applying various degrees of masking depending on the risk level of the subject and whether they are suspicious. The image information on the left of Figure 8 is the original image information of the subject that has been blurred, and the image in the upper middle is the blurring process applied when the subject is suspected of having criminal purposes, and the risk level is high. The image on the upper right shows the blurring process that is applied when the subject is not suspicious, and the risk level is high. The image in the lower middle shows a blurred image that is applied when the subject is suspicious, and the risk level is low. The image on the lower right shows a case where the subject is not suspicious, and the risk level is low.

**Figure 9:**
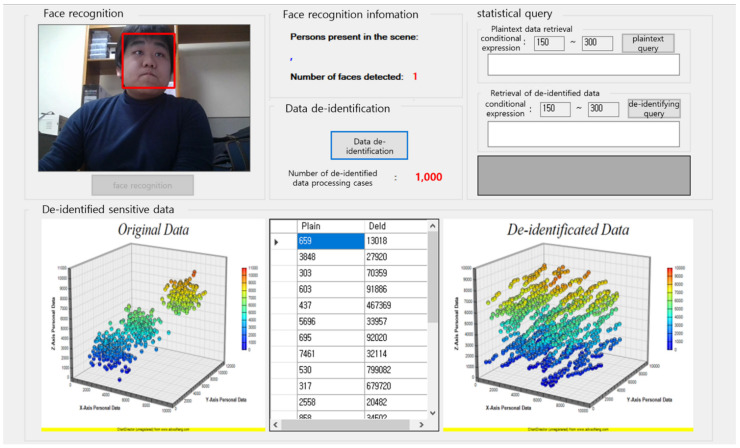
Three-dimensional graph of de-identification of facial area.

As described above, by varying the blurring difficulty depending on the risk level of the de-identified subject and whether he or she is suspected of having a criminal purpose, it is possible to acquire the information of the subject step by step. Figure 9 shows the system we created to visually represent the process of de-identification for each pixel based on the captured real-time image; it can be seen that the pixels are grouped together in a three-dimensional graph of the original information of the facial area in the image information. In the 3D graph of the de-identified image, the modulated values of the pixel values that were grouped together can be seen visually.

### 4.4. Performance of De-Identification Mechanisms and Analysis of Results

The de-identification mechanism we propose in this paper can prevent the indiscriminate leakage of information by de-identifying the image information, including the identification information and the facial area recorded in the database, converting it, and recording it as unidentifiable data.

**Figure 10:**
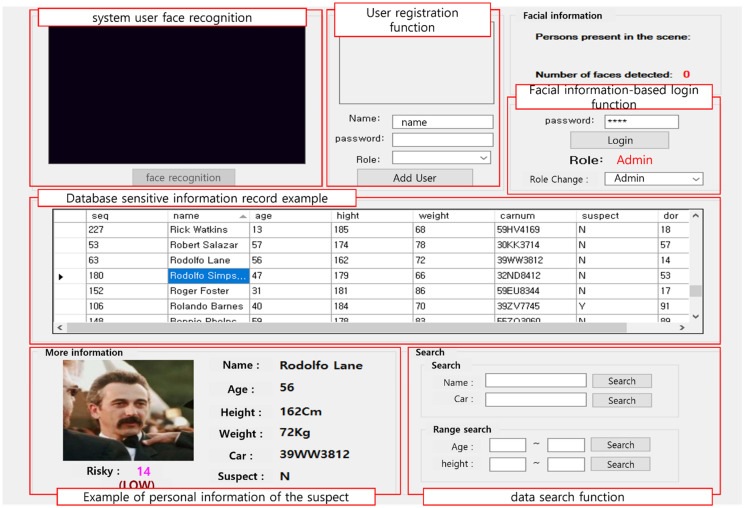
Visualization of the proposed mechanism.

This mechanism also restricts the information accessible to certain users by performing authentication based on the face area and a password and restricting access to information depending on the user’s role, making it impossible to identify original images containing a low-risk subject; conversely, high-risk subjects are de-identified in the image to an identifiable level by using the image information. Figure 10 shows a program running the face authentication function, and a function that provides user registration, de-identified information, and identification information to visualize its mechanism.

**Figure 11:**
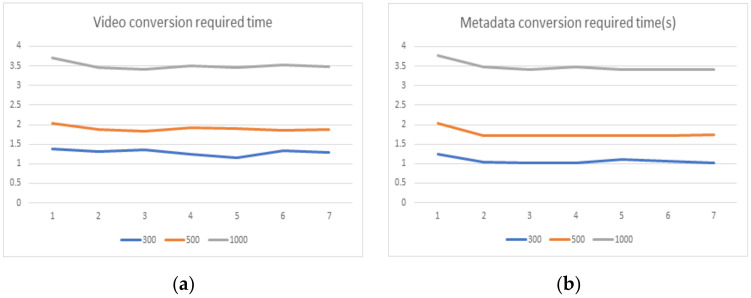
Time required for the proposed mechanisms: (**a**) time required for video conversion, (**b**) time required for metadata conversion.

Figure 11 shows the time required to proceed with the de-identification of video information and metainformation. Three hundred, five hundred, and one thousand conversion cases were performed, respectively. The metainformation conversion took an average of 1.076 s for 300 cases, 1.766 s for 500 cases, and 3.486 s for 1000 cases. The image information conversion took an average of 1.295 s for 300 cases, 1.892 s for 500 cases, and 3.5 s for 1000 cases.

In the case of applying the proposed mechanism to perform metainformation transformation through this performance result, it took about 3.6 ms per case to process 300 cases. In the case of 500 cases, it took about 3.53 ms, and in the case of 1000 cases, it took about 3.47 ms. As the number of cases increased, the execution speed per task decreased. When converting images, it took about 4.3 ms for 300 cases. In addition, it was confirmed that it took about 3.78 ms for 500 cases and about 3.5 ms for 1000 cases. Through this, it was confirmed that the performance speed per case for images was also inversely proportional to the number of cases. This shows that the greater the amount of information transformation required, the more effectively it can be processed.

## 5. Conclusions

Image information is information for identifying subjects, and it has a wide variety of utilization. This the area that is showing the biggest changes in the video surveillance system. Although the search for the subject by scanning and comparing the face image of the subject taken by the video surveillance system with the images in the database can be very effective, at the same time, it can be a privacy infringement. In a society that is becoming increasingly individualized, the leakage of privacy can become a major problem, and system administrators are obliged to ensure that recorded personal information is not leaked to the outside world. In particular, biometric information can become an obstacle to the construction of a smart city, because it may become difficult to use in the future due to a single leak.

The more thorough management of personal information recorded in the database is required, and many studies are underway on how to de-identify the information pertaining to the subject to prevent the leakage of personal information. In general, de-identified identification information produces a result value by hash calculation; therefore, it can have the same result at the same value. However, when de-identifying a subject, it may be difficult to identify the subject depending on the degree of de-identification.

In this paper, we proposed a method for de-identifying the identification information of a subject in the CCTV IoT environment based on the risk level of the subject and whether or not the subject is suspicious. The risk level of the subject can be set by the system administrator, and if the risk level of the subject is low, the information is less likely to be leaked to the outside world. However, if the risk level of the subject is high, the disclosure of the information may be requested for the management of the criminal. Furthermore, whether the subject is suspicious may affect the de-identification, in that it can serve as another criterion for identifying the subject. In this study, the indiscriminate leakage of privacy is prevented by varying the level of information disclosure according to the risk level. Such research can also be applied to the medical field as a method of discriminating data disclosure, such as allowing only the medical data required for each field to be disclosed. In the future, our research will move toward preventing data forgery by unauthorized users by using distributed structures such as blockchain to enhance the integrity of recorded data.

## Figures and Tables

**Table 1:** List of Interpol’s crime databases.

Group	Detailed Group
Individuals	Nominal data
	Child abusers and victims
Forensics	Fingerprints
	DNA profiles
	Facial recognition
Travel and official documents	Stolen and lost travel documents (SLTD)
	Stolen administrative documents (SAD)
	Counterfeit documents
	Comparison of genuine and fake documents
Stolen property	Motor vehicles
	Vessels
	Works of art
Firearms trafficking	Identification of firearms
	Tracing of firearms
	Comparison of ballistics data
Organized crime networks	Maritime piracy

**Table 2:** Separation of user roles in the suggestion mechanism.

Role	What They Do	Metadata Inquiry	Image Inquiry
Chief administrator	Manages with authority to create, modify, and/or delete data recorded in the system.	O	O
Investigator	Uses the system to solve cases based on information on suspects or missing persons recorded in the criminal database.	O	△
Server operator	Responsible for server maintenance as an on-site person for smooth execution of services.	△	△
Guest	Refers to the user who created the account in the video surveillance system and cannot access the data because the role is not assigned to the account accessed. Since the guest role is not granted access rights until another role is assigned to the subject, data access is not possible.	X	X

**Table 3:** Defined terms.

Abbreviations	Terms
G	Group by risk level
DR	Difficulty of risk-based de-identification
BR	Standard deviation depending on the subject’s charge
MaskB	Image compositing mask

## Data Availability

The data presented in this study are available on request from the corresponding author. The data are not publicly available due to the confidentiality pledge.

## References

[1] Bae S.Y., Chang P.-J. (2020). The effect of coronavirus disease-19 (COVID-19) risk perception on behavioural intention towards ‘untact’ tourism in South Korea during the first wave of the pandemic (March 2020). Curr. Issues Tour..

[2] Donghyeok L., Namje P. (2017). Electronic identity information hiding methods using a secret sharing scheme in multimedia-centric internet of things environment. Pers. Ubiquitous Comput..

[3] Namje P., Jung-Soo P., Hyoung-Jun K. (2015). Inter-Authentication and Session Key Sharing Procedure for Secure M2M/IoT Environment. Int. Inf. Inst. Inf..

[4] Stock L., Brown M., Bradley G. (2020). First Do No Harm With COVID-19: Corona Collateral Damage Syndrome. West. J. Emerg. Med. Integr. Emerg. Care Popul. Health.

[5] Donghyeok L., Namje P. (2017). A Secure Almanac Synchronization Method for Open IoT Maritime Cloud Environment. J. Korean Inst. Inf. Technol..

[6] Mwaffaq O., Nesreen O., Mohgammad A.A., Yousef E., Rudaina B. (2020). An IoT-based framework for early identification and monitoring of COVID-19 cases. Biomed. Signal Process. Control.

[7] Jinsu K., Namje P. (2019). Lightweight knowledge-based authentication model for intelligent closed circuit television in mobile personal computing. Pers. Ubiquitous Comput..

[8] Namje P., Namhi K. (2016). Mutual Authentication Scheme in Secure Internet of Things Technology for Comfortable Lifestyle. Sensors.

[9] Putri R.S., Purwanto A., Pramono R., Asbari M., Wijayanti L.M., Hyun C.C. (2020). Impact of the COVID-19 Pandemic on Online Home Learning: An Explorative Study of Primary Schools in Indonesia. Int. J. Adv. Sci. Technol..

[10] Namje P., Younghoon S., Youngsik J., Soo-Bum S., Chul K. (2018). The Analysis of the Appropriateness of Information Education Curriculum Standard Model for Elementary School in Korea. Studies in Computational Intelligence.

[11] Donghyeok L., Namje P. (2020). Blockchain based privacy preserving multimedia intelligent video surveillance using secure Merkle tree. Multimed. Tools Appl..

[12] Charles H.L., Anandh G.R., Patricia T.A., Chia-Shang J.L., Vishal P., John L.G., Paul E.K., Jay A. (2020). Virtual Read-Out: Radiology Education for the 21st Century During the COVID-19 Pandemic. Acad. Radiol..

[13] Marla B.K.S., Andrew C.S., Thierry A.G.M.H., Victor J.S. (2020). Response to the COVID-19 Pandemic: Practical Guide to Rapidly Deploying Home Workstations to Guarantee Radiology Services during Quarantine, Social Distancing, and Stay Home Orders. Med. Phys. Inform. Clin. Perspect..

[14] Donghyeok L., Namje P., Geonwoo K., Seunghun J. (2018). De-identification of metering data for smart grid personal security in intelligent CCTV-based P2P cloud computing environment. Peer Peer Netw. Appl..

[15] Jinsu K., Namje P., Geonwoo K., Seunghun J. (2019). CCTV Video Processing Metadata Security Scheme Using Character Order Preserving-Transformation in the Emerging Multimedia. Electronics.

[16] Zhe J., Minzheng D., Huizheng C., Wenxing Z., Teruyuki N., Makiko H., Bin C., Akihiro Y. (2020). Intercomparison between the aerosol optical properties retrieved by different inversion methods from SKYNET sky radiometer observations over Qionghai and Yucheng in China. Atmos. Meas. Tech..

[17] Che H., Shi G., Uchiyama A., Yamazaki A., Chen H., Goloub P., Zhang X. (2008). Intercomparison between aerosol optical properties by a PREDE skyradiometer and CIMEL sunphotometer over Beijing, China. Atmos. Chem. Phys..

[18] Jinsu K., Donghyeok L., Namje P. (2020). CCTV-RFID enabled multifactor authentication model for secure differential level video access control. Multimed. Tools Appl..

[19] Namje P., Byung-Gyu K., Jinsu K. (2019). A Mechanism of Masking Identification Information regarding Moving Objects Recorded on Visual Surveillance Systems by Differentially Implementing Access Permission. Electronics.

[20] Yiming L., Jie S., Shiyang C., Maja P. FT-RCNN: Real-time Visual Face Tracking with Region-based Convolutional Neural Networks. Proceedings of the 2020 15th IEEE International Conference on Automatic Face and Gesture Recognition (FG 2020).

[21] Namje P., Hyo-Chan B. (2014). Mobile middleware platform for secure vessel traffic system in IoT service environment. J. Secur. Commun. Netw..

[22] Lei Z., Zhang X., Yang S., Ren Z., Akindipe O.F. (2018). RFR-DLVT: A hybrid method for real-time face recognition using deep learning and visual tracking. Enterp. Inf. Syst..

[23] Jinsu K., Namje P. (2020). Blockchain-Based Data-Preserving AI Learning Environment Model for AI Cybersecurity Systems in IoT Service Environments. Appl. Sci..

[24] Toledo A.S.O., Carpi L.C., Atman A.P.F. (2020). Diversity Analysis Exposes Unexpected Key Roles in Multiplex Crime Networks. Springer Proc. Complex..

[25] Kamruzzaman M.M. (2020). E-crime Management System for Future Smart City. Adv. Intell. Syst. Comput..

[26] Donghyeok L., Namje P. (2017). Geocasting-based synchronization of Almanac on the maritime cloud for distributed smart surveillance. Supercomputing.

[27] Namje P., Jin K., Seungjoo K., Dongho W., Howon K. (2006). WIPI Mobile Platform with Secure Service for Mobile RFID Network Environment. Lect. Notes Comput. Sci..

[28] Interpol Our 18 Databases. https://www.interpol.int/How-we-work/Databases/Our-18-databases.

[29] Ling D., Wei Z., Huazhu F., Wenqi R., Xinpeng Z. (2019). An efficient privacy protection scheme for data security in video surveillance. J. Vis. Commun. Image Represent..

[30] Yifan W., Fan Y., Haibin L. (2018). Privacy-Protective-GAN for Face De-identification. arXiv.

[31] Alakh A., Rishika R., Pratik C., Lipo W. EPD-Net: A GAN-based Architecture for Face De-identification from Images. Proceedings of the 2020 IEEE International IOT, Electronics and Mechatronics Conference (IEMTRONICS).

[32] Namje P. (2018). The Core Competencies of SEL-based Innovative Creativity Education. Int. J. Pure Appl. Math..

[33] Agam B., Chandan G., Rana P.P. (2020). Optimizing the Implementation of COVID-19 “Immunity Certificates” Using Blockchain. J. Med. Syst..

[34] Namje P., Hongxin H., Qun J. (2016). Security and Privacy Mechanisms for Sensor Middleware and Application in Internet of Things (IoT). J. Distrib. Sens. Netw..

[35] Tornincasa S., Vezzetti E., Sandro M., Violante M., Marcolin F., Dagnes N., Ulrich L., Tregnaghi G. (2019). 3D Facial Action Units and Expression Recognition using a Crisp Logic. Comput. Sci. Comput.-Aided Des. Appl..

[36] Luigi M.G., Roberto D., Alberto L., Eliana D.G. (2016). Three-Dimensional Anthropometric Database of Attractive Caucasian Women: Standards and Comparisons. J. Craniofac. Surg..

